# Human treadmill walking needs attention

**DOI:** 10.1186/1743-0003-3-19

**Published:** 2006-08-21

**Authors:** Jean Philippe Regnaux, Johanna Robertson, Djamel Ben Smail, Olivier Daniel, Bernard Bussel

**Affiliations:** 1 Laboratoire d'Analyse du Mouvement, Hôpital R Poincaré 92380 Garches; APHP, UVSQ INSERM U731; UPMC-Paris 6, France; 2 University of California Los Angeles, Department of Neurology, 710 Westwood Plaza, Los Angeles, CA 90095, USA

## Abstract

**Background:**

The aim of the study was to assess the attentional requirements of steady state treadmill walking in human subjects using a dual task paradigm. The extent of decrement of a secondary (cognitive) RT task provides a measure of the attentional resources required to maintain performance of the primary (locomotor) task. Varying the level of difficulty of the reaction time (RT) task is used to verify the priority of allocation of attentional resources.

**Methods:**

11 healthy adult subjects were required to walk while simultaneously performing a RT task. Participants were instructed to bite a pressure transducer placed in the mouth as quickly as possible in response to an unpredictable electrical stimulation applied on the back of the neck. Each subject was tested under five different experimental conditions: simple RT task alone and while walking, recognition RT task alone and while walking, walking alone. A foot switch system composed of a pressure sensitive sensor was placed under the heel and forefoot of each foot to determine the gait cycle duration.

**Results:**

Gait cycle duration was unchanged (p > 0.05) by the addition of the RT task. Regardless of the level of difficulty of the RT task, the RTs were longer during treadmill walking than in sitting conditions (p < 0.01) indicating that an increased amount of resources are required for the maintainance of walking performance on a treadmill at a steady state. No interaction (p > 0.05) was found between the attentional demand of the walking task and the decrement of performance found in the RT task under varying levels of difficulty. This finding suggests that the healthy subjects prioritized the control of walking at the expense of cognitive performance.

**Conclusion:**

We conclude that treadmill walking in young adults is not a purely automatic task. The methodology and outcome measures used in this study provide an assessment of the attentional resources required by walking on the treadmill at a steady state.

## Background

A technique extensively used to study the neural processes involved in the control of walking in animals or in humans is walking on a treadmill [1]. This technique has also been proposed during the past decade as a training approach [2,3] to promote the recovery of locomotor function after a lesion of the central nervous system. It has been clearly demonstrated that non primate adult animals are able to "walk" and even adjust their gait patterns on a treadmill after transection of the spinal cord, for review see [4,5]. In this case, walking is not under any supraspinal control and is therefore completely automatic. The neural system responsible for this automatism in animals is thought to be located at spinal level and is referred to as the central pattern generator (CPG). This generator, which is activated by peripheral afferents (muscles, articulations and cutaneous), is thought to be stimulated through treadmill training [1]. This notion that the treadmill may stimulate automatic walking is, since the work of Barbeau et al [6], the underling rationale for its use in rehabilitation. However, in the intact animal, there is nothing to suggest that supraspinal control is not present. In contrast to the abundance of data gained from invertebrates, rats and cats, which has lead to the general assumption of a CPG underlying the central control of locomotion, there is relatively little known about spinal networks acting like CPGs in humans [7,8]. Contrary to the chronic adult spinalized cat, patients with a complete spinal cord injury (SCI) are unable to achieve a level of unassisted stepping. Most likely, cortical influences are stronger in human bipeds than mammalian quadrupeds [9]. This could mean that supraspinal processes are more predominant in humans than in cats [1,5,10]. Therefore, this raises the question regarding human locomotion on a treadmill: In healthy adults, is walking on a treadmill at a steady state an 'automatic' motor task or is it under voluntary control?

A common method used to quantify the automaticity of motor skills is the dual-task paradigm [11]. Dual task performance involves the execution of a primary task, which is the major focus of attention, and a secondary task performed at the same time [12]. If the two tasks can be performed as well simultaneously as separately, then at least one task seems to be automatic. On the other hand, if one task (e.g. walking) is performed less well when it is combined with the other task (e.g. talking), then both tasks must be non-automatic [13]. Performance decrement in the secondary task as a result of the simultaneous performance of a primary task is termed a dual-task interference effect. Hence, the extent of the decrement in the secondary task when performed with the primary task compared to when performed alone provides a measure of the attentional demands (cognitive regulation) of the primary task [14-16]. The interdependence between cognition and locomotor control is complex and depends on numerous factors, for a review see [17,18]. These factors include: the difficulty of the motor task [19], the type of cognitive task used [17], the age of the individual [11,19] and the resource allocation to a task in order to maintain performance at a certain level [20,21]. For example, Li and colleagues [20] investigated the link between sensorimotor (walking over the ground) and cognitive (memorizing) performance in younger and older adults when task difficulty was manipulated. They demonstrated that although older adults showed significant effects of divided attention in the cognitive domain, the attentional cost for walking was comparable for the two age groups. This finding is related to the issue of task priority [21,22], in that walking and maintaining balance control are prioritized at the expense of cognitive performance. Only a few studies [12,23] have investigated the degree of interference between steady state walking on a treadmill and a cognitive task. The amount of attention required to walk on a treadmill has not been previously examined and is the topic of this investigation.

To quantify the attentional resources allocated to walking on the treadmill at a steady state, we used a dual-task paradigm in which the secondary task was a reaction time (RT) task performed at two levels of difficulty. One measure proposed among the cognitive tasks used to evaluate the attentional cost of a locomotor task, is reaction time [24-26]. While the attention resources model is always invoked in the interpretation of a dual task interference [15,17], some studies [27,28] show that modifications of a postural task can be provoked by respiration (structural interference) and not caused by competing demands for limited attentional resources (capacity interference). For this reason, we chose an unusual RT task for which the response modality was biting on a pressure sensor. This modality has no respiratory involvement (ex. vocal response) or motor action of the upper limb (ex. push button) for which we could suppose that there are shared execution pathways with locomotion [5,29].

The aim of the present study was to determine the amount of resources allocated to the control of steady state treadmill walking in healthy adult subjects. By varying the RT task difficulty, we wanted to make sure that attentional resources were allocated, in priority, to the control of the walking task.

## Methods

### Subjects

The subjects in this study were eleven healthy adults (mean age: 25.3 years, range: 22–37) with no known neurological, orthopaedic or cognitive impairments. All the subjects were graduate students who were familiar with treadmill walking. Informed consent was obtained from each subject prior to their participation in the study. All procedures were performed with the approval of the local ethics committee and complied with the standards defined in the Declaration of Helsinki (2000).

### Tasks

Each subject was asked to walk steadily on a treadmill without holding on to the handrail and while looking straight ahead at a wall that was approximately 3 meters away. Self-adopted walking speed over the floor (measured with a stopwatch on a 10 m walkway) was used as the basis for calculation of preferred treadmill speed for each subject. The treadmill speed was maintained constant during the trial. A foot switch system composed of a pressure sensitive sensor (Interlink model FSR 151) was placed under the heel and forefoot of each foot to determine the gait cycle duration. The gait cycle was defined as initial heel contact of one foot to the next initial contact of that same foot. All signals were digitalized at 1000 Hz, transmitted to a PC.

The secondary RT task consisted of biting a pressure transducer (Interlink model FSR 151) placed in the mouth in response to an unpredictable electrical stimulation (single stimulus, duration: 10 ms) applied by an electrode on the back of the neck. This modality was chosen so that the response pathways would be as independent as possible from the motor pathways of locomotion. The perception threshold of the stimulation was determined for each subject and before each experimental condition. The stimulus intensity was then doubled to ensure that it was clearly perceived and remained constant during the trial. Stimuli were manually triggered by an examiner at a frequency ranging from 2,000 to 5,000 ms.

Two levels of difficulty of the RT task were used. The *simple RT task *consisted of responding to a single stimulus as rapidly as possible. The *complex RT task *consisted of a recognition RT. Stimuli of different strength (weak, strong) were individually determined for each subject. Subjects were instructed to bite the pressure transducer placed in the mouth as rapidly as possible only when a weak stimulus was presented. If subjects did not adhere to these instructions, the response was considered to be an error. The number of errors for each condition was noted for each subject.

### Procedure

We assessed the attentional requirements for the control of steady state treadmill walking according to the principles of the dual task paradigm [30]. According to this paradigm, subjects perform a secondary cognitively demanding task while engaged in a primary task. When attention is focused on the primary task, the amount of attentional resources allocated to that task increases in order to maintain or increase performance. Such an increase in allocation of attention leads to depletion of the resources devoted to the other, non priority task [14]. Therefore, disruption in the performance of the secondary task is regarded as a probe to evaluate the attentional resources needed to preserve or improve performance of the primary motor task [14,19]. In our study, the secondary task was the RT task and the primary task was to walk at steady state on the treadmill.

Each subject was tested under five different experimental conditions: simple RT task alone and while walking, recognition RT task alone and while walking, walking alone. Single-task refers to control conditions where either RT task or walking is assessed alone and dual-task, to experimental conditions where RT task and walking are performed simultaneously. In single task cognitive conditions, subjects performed the RT tasks while seated, as a baseline measurement for the performance of the secondary task. To ensure that subjects did not neglect walking performance at the expense of the RT task performance, participants were also submitted to a control condition in which they were simply asked to walk on the treadmill without performing any additional task. All conditions were presented in random order across subjects.

At the onset of each session, subjects practiced walking on the treadmill at a comfortable speed for 10 minutes. 20 RT stimuli were delivered systematically at the start of each trial to enable the subjects to familiarise themselves with the conditions of the RT task. Visual verification of the stability of the RT was carried out before beginning recording.

### Statistical analysis

The RT was defined by the interval between the electrical stimulus and the start of the response (biting the pressure sensor). For each condition, all reaction times longer than 100 ms were accepted. 30 accepted RTs were necessary to constitute a trial.

To determine whether the walking task performance was affected by the addition of the RT task, the gait cycle duration was compared between the "control" (without the RT tasks) and the experimental (with the RT tasks) walking conditions using a one-way analysis of variance (ANOVA) with repeated measures.

To determine the effects of task conditions (sitting vs walking on the treadmill), difficulty of the RT task (simple vs complex) on the RT task performance and associated interaction, a two-way analysis of variance with repeated measures was used.

The within-subjects variability was neutralized by a blocking design technique [31] for all the statistical analysis. The normality assumption was checked by examining the residuals. The limit of significance was defined at 0.05

## Results

### Effect of the cognitive task on treadmill walking performance

The duration of the gait cycle for each subject in the three conditions: walking, walking with simple RT, walking with complex RT are reported in table 1. No differences were observed for the gait cycle duration between the three walking conditions (F(2, 16) = 0.90; p > 0.05). This result indicates that the walking task performances were not modified under the dual task conditions compared with the single task condition and confirmed our operational assumption that the treadmill walking task remained the primary task throughout the experiment.

**Table 1:** Individual characteristics of the gait cycle step duration in 9 healthy subjects in different walking conditions on the treadmill. 2 subjects were omitted for technical reasons

**Subject Number**	**Treadmill speed (Km/h)**	**WTdM alone**	**WTdM with simple RTs**	**WTdM with complex RTs**
1	3,6	1167 ms	1154 ms	1183 ms
2	4,3	1192 ms	1209 ms	1213 ms
3	3,8	1244 ms	1235 ms	1226 ms
4	4,3	1062 ms	1077 ms	1067 ms
5	5	1114 ms	1100 ms	1115 ms
6	3,4	1254 ms	1236 ms	1216 ms
7	4,1	1324 ms	1296 ms	1287 ms
8	3,3	1186 ms	1193 ms	1245 ms
9	3,1	1233 ms	1235 ms	1296 ms

mean	-	1197,33 ms	1192,78 ms	1205,33 ms
SD	-	78,30	70,60	74,88
CV	-	6,54	5,92	6,20

Abbreviations: SD = standard deviation, CV = coefficient of variation, WTdM = walking on the treadmill.

### The attentional demands on steady state treadmill walking

RT data were examined under the 2 experimental conditions (sitting vs walking on the treadmill) x 2 levels of difficulty of the RT (simple vs complex) repeated measures ANOVA. Results showed main effects of experimental conditions (F(1, 30) = 7.36, p < 0.01) and levels of difficulty of the RT (F(1, 30) = 352.87, p < 0.001) but there was no significant interaction between the main factors (F(1, 30) = 0.52, p = 0.48).

The mean (±SD) RTs measured during sitting and walking on the treadmill were 225 ± 36 ms and 259 ± 45 ms under simple condition; 419 ± 63 ms and 439 ± 52 ms under complex condition. The performance in the RT task decreased under the complex condition confirming that the difficulty in the RT task was explicitly manipulated. The RTs were significantly longer when walking on the treadmill than when sitting, suggesting that some additional attentionnal resources were allocated to the treadmill walking task.

**Figure 1:**
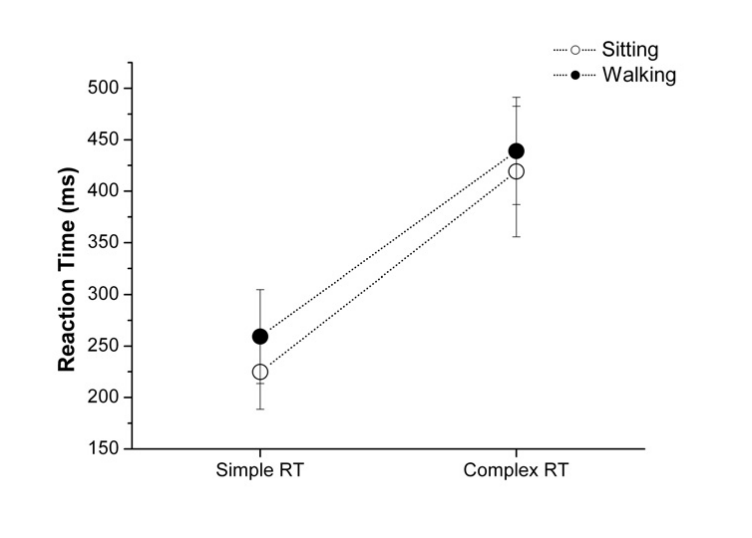
Mean reaction times and standard error (SE) as a function of difficulty for sitting (white circle) and stabilised walking on the treadmill (black circle) in eleven healthy subjects.

The absence of an interaction between the experimental conditions and difficulty of the RT task indicates that the significant difference in RT between the walking and sitting conditions was similar for the simple and the complex RT task conditions (Fig 1).

## Discussion

The aim of this study was to determine, using a dual task paradigm, whether the control of treadmill walking in healthy subjects requires attentional resources. Results of the present experiment clearly show longer RTs during the walking condition than during the sitting conditions regardless of the level of difficulty of the RT task.

### Possible causes of dual-task interference

The increase of the RTs observed during the walking task may be explained by the theory of attention related to a capacity model where resources are shared [21,30,32,33]. In the resource framework, dual task interference could be attributed to either structural or capacity limitations [34]. Interference between structures can occur when two tasks share the same perceptual or executive pathways.

The experimental design we used was chosen *deliberately *to ensure that the response pathways for each task were as independent as possible [30]. We believe that is improbable that the two tasks shared the same response pathways for two reasons. First, we found no changes in gait parameters between the simple and the dual task conditions [27,28]. Secondly, the finding that the greatest dual task interference occurred with the complex RT task condition rather implies that the two tasks compete for the same limited resources [21,35]. If the interference was related to structure, performances of the RT task would be similar while walking for the simple and complex RT task conditions [34]. Furthermore, in a previous study using the same design [36], we reported specific changes in RT task performance which were related to the phase of the gait cycle. Young adults demonstrated longer RTs during the double limb support phases than during the single limb support phases when walking at a steady state on the treadmill. It seems highly unlikely that structural limitation would occur at certain instants and not others.

An alternative explanation for the findings we present, however, is that the observed changes in RT are unrelated to the information processing demands associated with the control of walking on the treadmill, and that, instead, decreased RT task performance exemplifies the general attenuation of afferent signals associated with Pieron's law. Evidence in many studies suggests that our ability to detect signals from sensory stimulation is reduced during movement [37-39]. Pieron's law dictates that RT decreases in a hyperbolic fashion with increased stimulus intensity, regardless of the difficulty of the RT task [40,41]. Following this law, the weaker the stimulus, the longer the reaction time. Consequently, if the movement induced a general attenuation of the perception of the stimulus intensity, then the elevated RTs may not be indicative of changes in the attentional demands associated with gait, but instead, may reflect a reduction in perception of the strength of the stimulation.

In the current study, stimulus intensity was reset before each condition; at levels twice that of the level of initial perception in order that the subjects could clearly perceive it 100% of the time. It remained constant in each condition and moreover, was always higher during the walking condition than in the sitting condition. We therefore believe that the interference did not occur at the stimulus perception stage.

Thus, following the principles of the dual task paradigm [30], we interpreted the observed changes in RT to reflect the attentional demands associated with walking on a treadmill.

### The cognitive load of walking on a treadmill

Our young adult subjects demonstrated longer RTs during walking than during the sitting condition; performing the RT task did not affect their gait patterns. These findings confirm those of Abernethy et al [12], demonstrating that steady state treadmill walking requires some attentional resources and suggests that locomotor control is not completely automatised.

It is interesting that increasing the difficulty of the RT task had no impact on performance of the walking task. According to resource theories [21], performance decrement observed in the RT task in which difficulty is explicitly manipulated is considered to demonstrate resource allocation [14,18,20,22,42,43] in order to meet the primary task demand. This pattern suggests that subjects prioritize walking performance at the expense of any secondary information processing task and provides an independent index of the attentional resources required by walking on the treadmill at a steady state.

### Limitations

The longer RT in the dual task condition might be specific to the subjects' ability to walk on a motor driven treadmill. It is possible that treadmill walking requires attentional resources because of the necessity of dynamic balance control linked to the use of the treadmill, as has been recently suggested by Grabiner and Troy [44].

However, the voluntary control demonstrated in steady state treadmill walking could also be a reflection of voluntary control required in walking on the ground. As claimed by Nielsen [10], the supra spinal structures which contribute to voluntary modifications of the gait pattern are also implicated in the control of human walking. This claim warrants further investigation by comparison of walking on a treadmill and on the ground under a cognitive load.

In summary, treadmill walking in young adults requires attentional resources and is not a purely automatic task. Gait parameters were not modified by the addition of a RT task or according to the level of difficulty of the RT task indicating that the subjects prioritized the control of walking at the expense of cognitive performance. The methodology and outcome measures used in this study provide an assessment of the attentional resources required for steady state treadmill walking.

## Competing interests

The author(s) declare that they have no competing interests.

## Authors' contributions

JP Regnaux was the main investigator of the study, participated in the design, evaluated the data and results, performed the statistical analysis and wrote the manuscript. J Robertson helped to translate and was involved in drafting the manuscript. D Ben Smail was involved in collecting and in interpreting the data. O Daniel was involved in collecting and in interpreting the data. B Bussel participated in the design and was involved in drafting and revising the manuscript.

All authors read and approved the final manuscript
